# Nuclear receptor 4A1 (NR4A1) as a drug target for treating rhabdomyosarcoma (RMS)

**DOI:** 10.18632/oncotarget.9112

**Published:** 2016-04-29

**Authors:** Alexandra Lacey, Erik Hedrick, Xi Li, Ketan Patel, Ravi Doddapaneni, Mandip Singh, Stephen Safe

**Affiliations:** ^1^ Department of Veterinary Physiology and Pharmacology, Texas A&M University, College Station, 77843, TX, USA; ^2^ College of Pharmacy and Pharmaceutical Sciences, Florida A&M University, Tallahassee, 32307, FL, USA

**Keywords:** NR4A1, C-DIMs, NR4A1 antagonists, rhabdomyosarcoma, inhibition

## Abstract

The orphan nuclear receptor NR4A1 is expressed in tumors from rhabdomyosarcoma (RMS) patients and Rh30 and RD RMS cell lines, and we used RNA interference (RNAi) to investigate the role of this receptor in RMS cells. Knockdown of NR4A1 in Rh30 cells decreased cell proliferation, induced Annexin V staining and induced polyADPribose polymerase (PARP) cleavage and these results were similar to those observed in other solid tumors. Previous studies show that NR4A1 regulates expression of growth promoting/pro-survival genes with GC-rich promoters, activates mTOR through suppression of p53, and maintains low oxidative stress by regulating expression of isocitrate dehydrogenase 1 (IDH1) and thioredoxin domain containing 5 (TXNDC5). Results of RNAi studies demonstrated that NR4A1 also regulates these pathways and associated genes in RMS cells and thereby exhibits pro-oncogenic activity. 1,1-Bis(3-indolyl)-1-(*p*-substituted phenyl)methane (C-DIM) analogs containing *p*-hydroxyl (DIM-C-pPhOH) and *p*-carboxymethyl (DIM-C-pPhCO_2_Me) substituents are NR4A1 ligands that decreased NR4A1-dependent transactivation in RMS cells and inhibited RMS cell and tumor growth and induced apoptosis. Moreover, the effects of NR4A1 knockdown and the C-DIM/NR4A1 antagonists were comparable as inhibitors of NR4A1-dependent genes/pathways. Both NR4A1 knockdown and treatment with DIM-C-pPhOH and DIM-C-pPhCO_2_Me also induced ROS which activated stress genes and induced sestrin 2 which activated AMPK and inhibited mTOR in the mutant p53 RMS cells. Since NR4A1 regulates several growth-promoting/pro-survival pathways in RMS, the C-DIM/NR4A1 antagonists represent a novel mechanism-based approach for treating this disease alone or in combination and thereby reducing the adverse effects of current cytotoxic therapies.

## INTRODUCTION

Rhabdomyosarcoma (RMS) is the most common soft tissue sarcoma that is primarily observed in children and adolescents and accounts for 5% of all pediatric cancers and 50% of soft tissue sarcomas in children [1, 2]. Embryonal RMS (ERMS) and alveolar RMS (ARMS) are the two major classes of RMS in children and adolescents and differ with respect to their histology, genetics, treatment, and prognosis [1–4]. ERMS accounts for over 60% of RMS patients and is associated with loss of heterozygosity at the 11p15 locus. ERMS patients have a favorable initial prognosis; however, the overall survival of patients with metastatic ERMS is only 40% [3]. ARMS occurs in a lower percentage of RMS patients and is associated with translocations resulting in formation of pro-oncogenic gene products resulting from the fusion of PAX3 or PAX7 with the Forkhead gene *FOXO1A* [5, 6]. ARMS patients have a poor diagnosis and patient survival is < 10% for metastatic ARMS.

RMS patients are treated with radiotherapy, surgery, and chemotherapy using cytotoxic drugs and/or drug combinations, and successful treatment varies with tumor type (ARMS vs. ERMS) and extent of metastasis. However, a recent study on adults treated for childhood cancers showed that over 90% of these individuals exhibited chronic adverse health conditions later in life [7], demonstrating that there is a critical need for development of new mechanism-based drugs for treatment of RMS.

**Figure 1:**
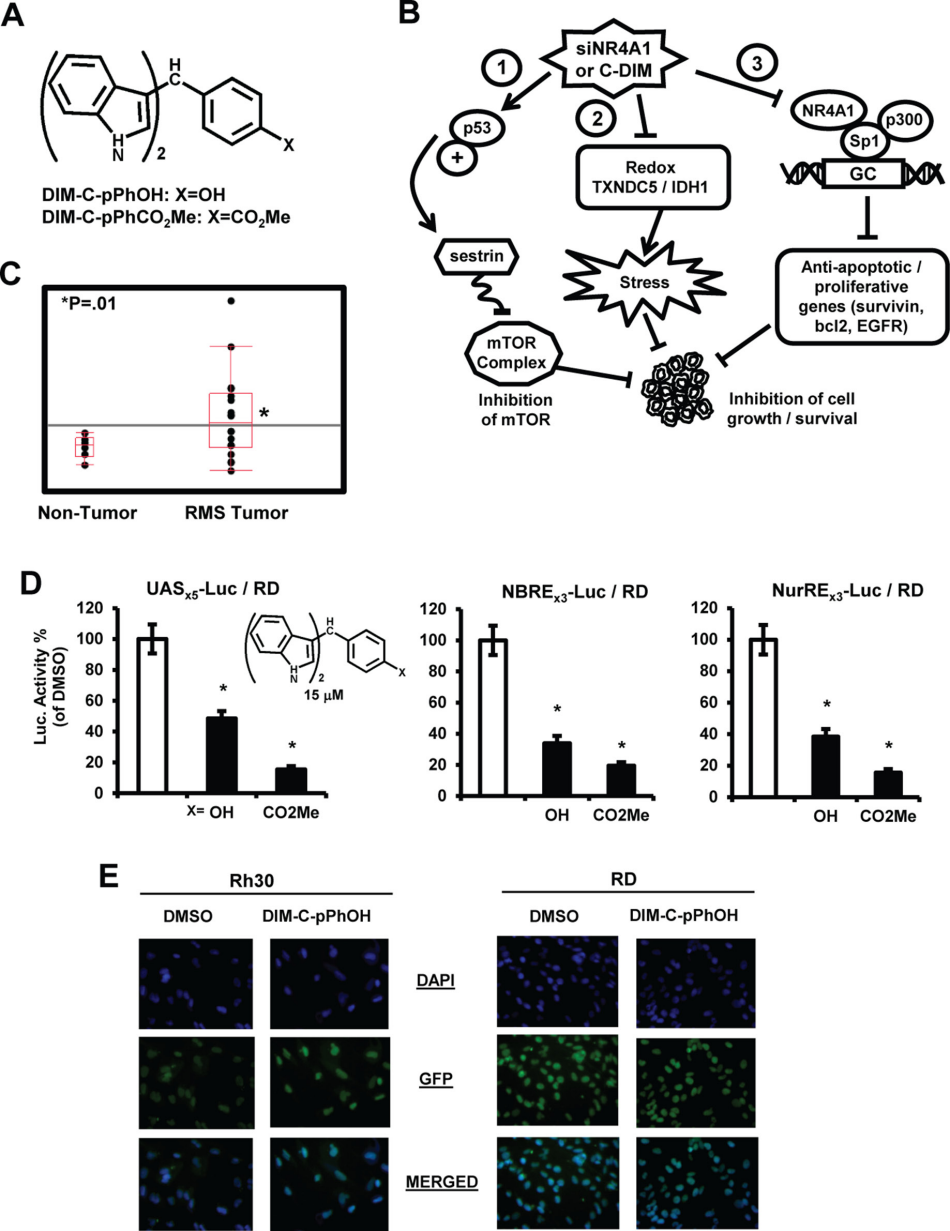
NR4A1 expression and transactivation by C-DIMs (**A**) Structure of C-DIMs and (**B**) NR4A1-regulated pro-oncogenic pathways in cancer cells. (**C**) Analysis of NR4A1 gene expression in patient-derived mRNA acquired from the NCBI GEO dataset GSE2851. (**D**) C-DIMs inhibit NR4A1-dependent transactivation. RD cells were transfected with pGAL4-NR4A1/UAS_x5_-luc, NBRE_x3_-luc or NuRE_s3_-luc, treated with DMSO or 15 μM DIM-C-pPhOH or DIM-C-pPhCO_2_Me, and luciferase activity was determined as outlined in the Materials and Methods. Results are expressed as means ± SE for at least 3 separate experiments and significantly (*p <* 0.05) decreased activity is indicated (*). (**E**) Cellular localization of NR4A1. Rh30 (A) and RD (B) cells were treated with DMSO or 20 μM DIM-C-pPhOH for 24 hr and cells were stained with DAPI and a fluorescent NR4A1 antibody. The individual and merged staining was determined as outlined in the Materials and Methods.

The orphan nuclear receptor 4A1 (NR4A1, Nur77/TR3) does not have an endogenous ligand; however, this receptor plays a key role in cellular homeostasis and in several diseases including cancer [8, 9]. NR4A1 is overexpressed in lung, breast, pancreatic and colon cancer patients [9–13], and functional studies show that NR4A1 is pro-oncogenic and plays a role in cancer cell proliferation, survival, migration and invasion [reviewed in 9]. Several structurally-diverse ligands that directly bind NR4A1 have been characterized [14–17] and studies in this laboratory have shown that among a series of 1,1-bis(3-indolyl)-1-(*p*-substituted phenyl)methanes (C-DIMs), several compounds including the *p*-hydroxy (DIM-C-pPhOH) and *p*-carbomethoxy (DIM-C-pPhCO_2_Me) analogs directly bind NR4A1 (Figure 1A). Results of RNA interference (RNAi) studies show that NR4A1 activates mTOR by binding and inactivating p53 [12], regulates genes such as isocitrate dehydrogenase 1 (IDH1) and thioredoxin domain-containing 5 (TXNDC5) to decrease cellular stress [18], and regulates expression of growth promoting/survival genes such as survivin and epidermal growth factor receptor (EGFR) through NR4A1-Sp1 interactions with their proximal GC-rich promoter elements [19]. The pro-oncogenic NR4A1-regulated activities have previously been characterized in colon, lung and pancreatic cancer cells [12, 16, 18, 19], and the C-DIM/NR4A1 antagonists inhibited these pathways (Figure 1B) and gave results comparable to those observed for RNA interference (RNAi). In preliminary data mining studies, we observed that NR4A1 was also overexpressed in RMS tumors compared to normal tissue and high levels were observed in prototypical ARMS (Rh30) and ERMS (RD) cell lines. This study also demonstrates that NR4A1 regulates pro-oncogenic pathways (Figure 1B) in RMS cells and C-DIM/NR4A1 antagonists inhibit these responses, demonstrating that NR4A1 is a potential novel target for RMS chemotherapy.

## RESULTS

### NR4A1 expression and transactivation

Examination of publically-available RMS array data show that NR4A1 mRNA is more highly expressed in RMS tumors compared to non-tumor tissue (Figure 1C). Previous studies show that the C-DIM compounds DIM-C-pPhOH and DIM-C-pPhCO_2_Me bind NR4A1 and act as NR4A1 antagonists for transactivation assays in colon cancer cells [16] and therefore these compounds were also used in this study on RMS cells. RD cells were transfected with constructs containing the DNA binding domain of the yeast GAL4 protein fused to NR4A1 and the UAS_X5_ luc construct containing 5 GAL4 response elements, and treatment with DIM-C-pPhOH or DIM-C-pPhCO_2_Me decreased luciferase activity (Figure 1D). DIM-C-pPhOH and DIM-C-pPhCO_2_Me also decreased luciferase activity in RD cells transfected with NBRE_3_-luc and NuRE_3_-luc constructs containing 3 binding sites for NR4A1 monomer and homodimer, respectively (Figure 1D). Basal activity was low for both constructs but significantly enhanced by cotransfection with a FLAG-TR3 expression plasmid in RD cells. These results were comparable to those previously observed in colon cancer cells [16] and demonstrate that the two C-DIM compounds exhibit antagonist activity for transactivation in RD cells. Immunostaining of Rh30 and RD cells with DAPI and NR4A1 antibodies showed that NR4A1 was nuclear in these RMS cell lines (Figure 1E). Moreover, the u = mu (micro) after treatment with 20 uM DIM-C-pPhOH for 24 hr, we did not observe any nuclear export of NR4A1 which was comparable to observations in other cancer cell lines [12, 16, 18, 19].

### Role of NR4A1 in RMS cell growth and survival

**Figure 2:**
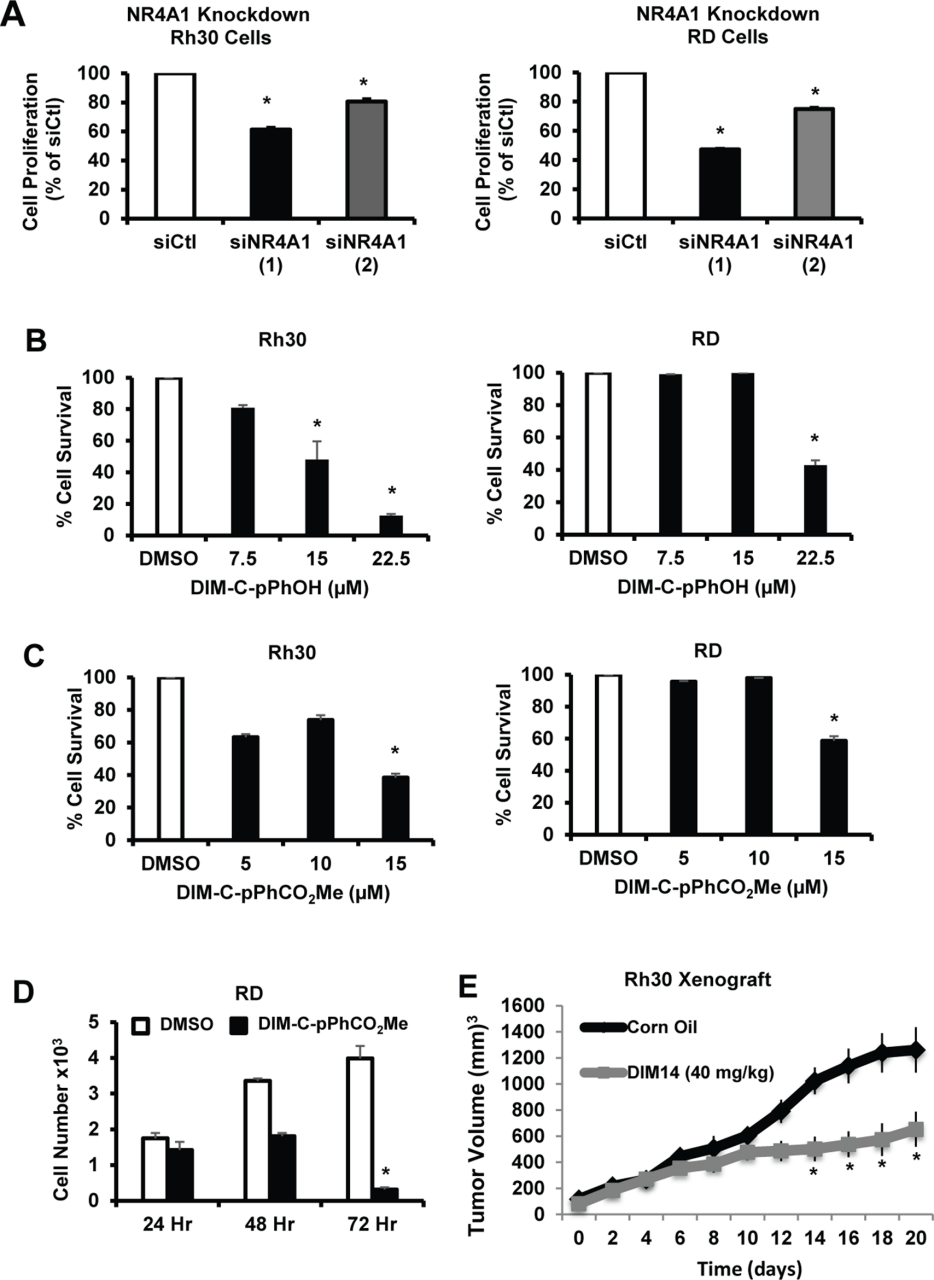
NR4A1 regulates growth of RMS cells which can be inhibited by C-DIM/NR4A1 antagonists (**A**) Rh30 and Rd cells were transfected with two different oligonucleotides targeted to NR4A1 [siNR4A1(1) and siNR4A1(2)], and after 72 hr, the cells were counted and compared to the number of cells observed after transfection with a non-specific control (siCtl) oligonucleotide. Rh30 and RD cells were treated with different concentrations of DIM-C-pPhOH (**B**) or DIM-C-pPhCO_2_Me (**C**) for 24 hr, and (**D**) RD cells were treated with 15 μM DIM-C-pPhCO_2_Me for 24, 48 or 72 hr. Cells were counted and compared to the number observed after treatment with the solvent control (DMSO, set at 100%). (**E**) In a preliminary *in vivo* study, we observed that after treatment of athymic nude mice with DIM-C-pPhOH (40 mg/kg/d for 28 days), there was a small but significant inhibition of tumor growth and future studies will use a higher dose of this compound. Results (A – E) are expressed as means ± SE for at least 3 separate treatments for each group and significant (*p <* 0.05) growth inhibition is indicated (*).

**Figure 3:**
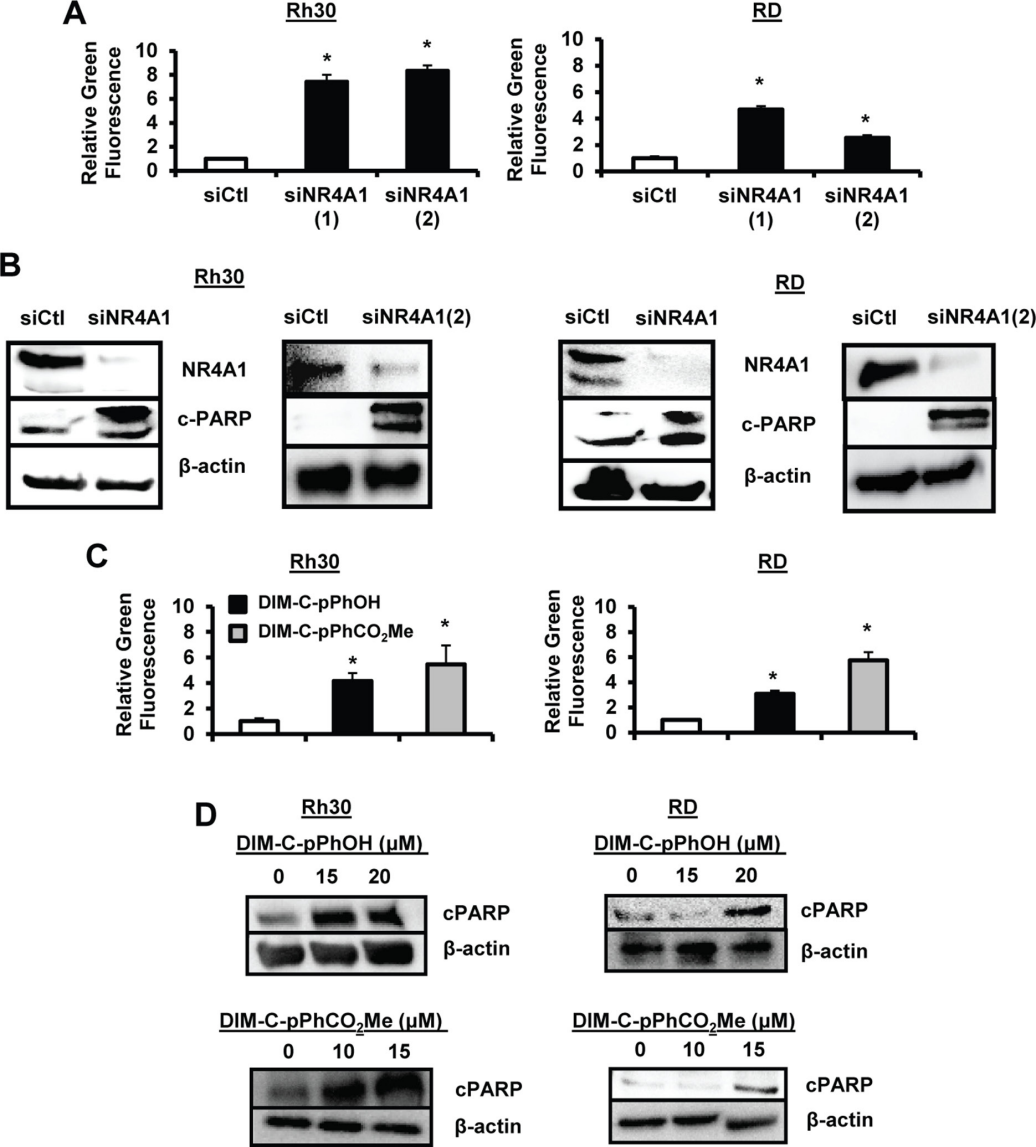
NR4A1 regulates RMS survival that can be inhibited by C-DIM/NR4A1 antagonists Rh30 and RD cells were transfected with siNR4A1 or siNR4A2, and induction of Annexin V staining (**A**) or enhanced PARP cleavage (**B**) were determined as outlined in the Materials and Methods. Rh30 and RD cells were treated with DIM-C-pPhOH or DIM-C-pPhCO_2_Me and effects on Annexin V staining (**C**) or enhanced PARP cleavage (**D**) were determined as outlined in the Materials and Methods. Results (A and C) were expressed as means ± SE for at least 3 replicate determinations per treatment group and significant (*p <* 0.05) induction is indicated (*).

Transfection of Rh30 and RD cells with siNR4A1 significantly decreased proliferation of Rh30 and RD cells and comparable results were observed for two different siRNAs (Figure 2A). Treatment of Rh30 cells with 7.5 to 22.5 μM DIM-C-pPhOH and 5 to 15 μM DIM-C-pPhCO_2_Me of the NR4A1 antagonists for 24 hr also inhibited growth of RH30 (Figure 2B) and RD (Figure 2C) cells with IC_50_ values ranging from 6.6 to 29 μM. Figure 2D also shows that although inhibition of RD cell growth after treatment with 15 μM DIM-C-pPhCO_2_Me was only 20–25%, after prolonged treatment (48 and 72 hr), more complete growth inhibition was observed. In addition, we also observed that DIM-C-pPhOH (40 mg/kg/d) inhibited tumor growth in athymic nude mice bearing Rh30 cells as xenografts (Figure 2E). We also investigated the role of NR4A1 in mediating survival of Rh30 and RD cells, and Figure 3A shows that transfection of these cells with siNR4A1 resulted in the induction of Annexin V staining. Moreover, transfection of Rh30 and RD cells with siNR4A1 also induced PARP cleavage, another marker of apoptosis in these cells (Figure 3B). Treatment of Rh30 and RD cells with the NR4A1 antagonists DIM-C-pPhOH and DIM-C-pPhCO_2_Me also induced Annexin V staining (Figure 3C) and PARP cleavage (Figure 3D), thus confirming the pro-survival activity of NR4A1 in RMS cells and effects of C-DIM/NR4A1 antagonists as inhibitors of cell growth and survival.

### NR4A1 antagonists inhibit growth/survival pathways and gene products in RMS cells

**Figure 4:**
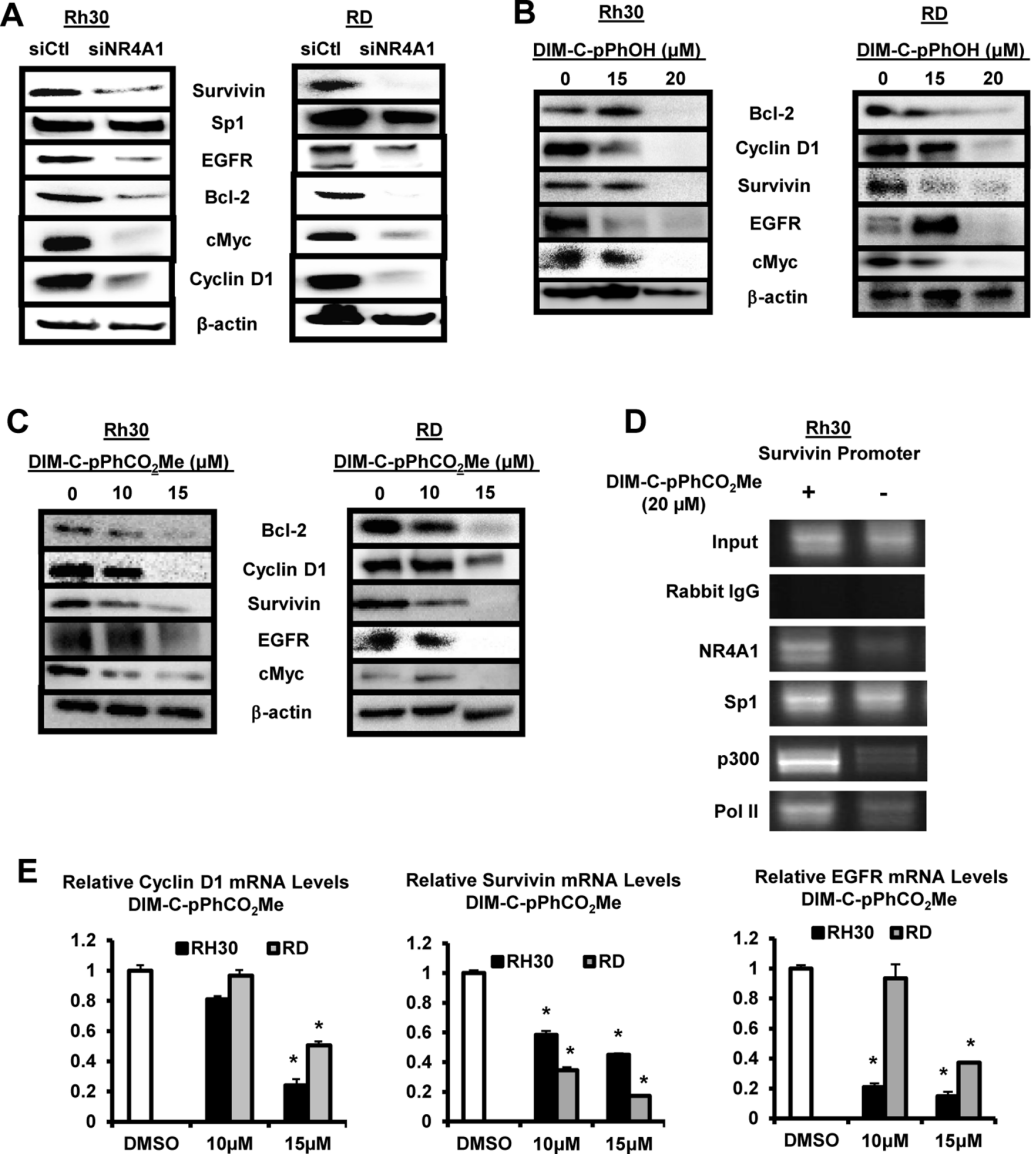
NR4A1 regulation of pro-survival/growth promoting genes and their inhibition by C-DIM/NR4A1 antagonists (**A**) Rh30 and RD cells were transfected with siNR4A1 or siCtl, and whole cell lysates were analyzed by Western blots as outlined in the Materials and Methods. Rh30 and RD cells were treated with DMSO (solvent control), DIM-C-pPhOH (**B**) or DIM-C-pPhCO_2_Me (**C**), and whole cell lysates were analyzed by Western blot as outlined in the Materials and Methods. (**D**) Rh30 and RD cells were treated with DIM-C-pPhCO_2_Me, and binding of NR4A1, Sp1, p300 and pol II to the survivin promoter was determined in a ChiP assay. (**E**) Cells were treated with DIM-C-pPhCO_2_Me, and survivin, cyclin D1 and EGFR mRNA levels were determine by real time PCR. Results are expressed as means ± SE (3 replicates) and significant (*p <* 0.05) changes in gene expression are indicated (*).

Previous studies show that NR4A1 acts as a coactivator of genes with GC-rich promoters (Figure 1B, pathway 3) that play a role in cancer cell proliferation and survival, and these include *survivin*, *bcl-2*, *cyclin D1*, epidermal growth factor receptor (*EGFR*) and the oncogene c*Myc* [16, 19]. Knockdown of NR4A1 by RNA in Rh30 and RD cells decreased expression of several genes with GC-rich promoters including EGFR, bcl2, c-Myc and cyclin D1, and this was accompanied by minimal effects on expression of Sp1 (Figure 4A). Treatment of Rh30 and RD cells with the NR4A1 antagonists DIM-C-pPhOH (Figure 4B) and DIM-C-pPhCO_2_Me (Figure 4C) also decreased expression of survivin, bcl-2, cyclin D1, EGFR and cMyc, and these results paralleled those observed after knockdown of NR4A1 in these cells lines (Figure 4A). DIM-C-pPhCO_2_Me was used to further investigate the mechanism of downregulation of Sp-regulated genes at the transcriptional level. In a ChIP assay, DIM-C-pPhCO_2_Me decreased binding of NR4A1 and p300 (but not Sp1) at the GC-rich region of the survivin promoter and pol II binding was also decreased (Figure 4D). These results are comparable to previous studies in pancreatic cancer cells showing that p300/NR4A1 coregulated survivin expression by interacting with DNA-bound Sp1 (Figure 1B) [19]. In addition, DIM-C-pPhCO_2_Me also decreased expression of survivin, cyclin D1 and EGFR mRNA levels (Figure 4E). Thus, NR4A1 also coregulates expression of Sp-regulated pro-survival/growth promoting genes with GC-rich promoters in RMS cells.

**Figure 5:**
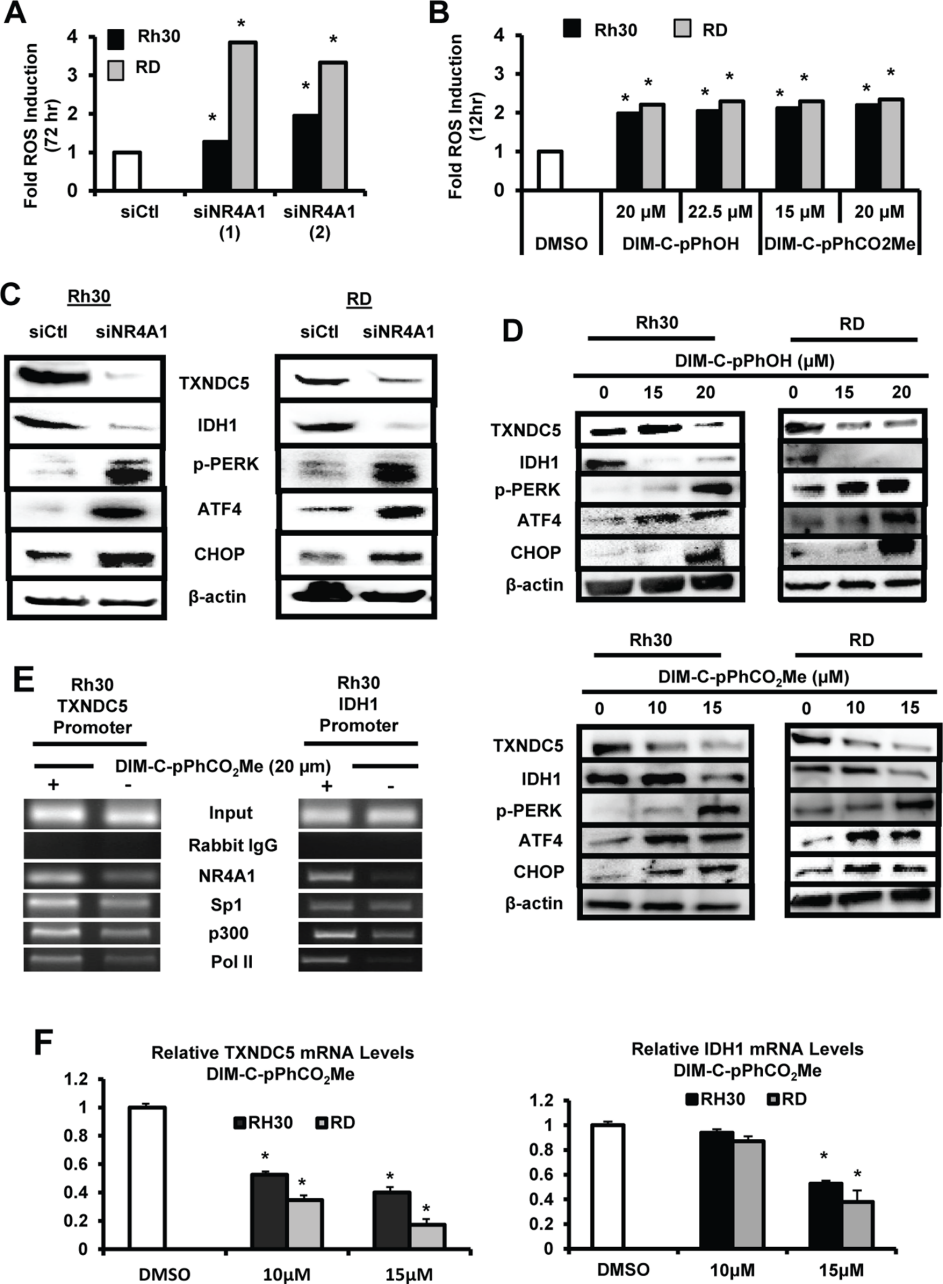
Role of NR4A1 in regulating oxidative stress Rh30 and RD cells were either transfected with siNR4A1(1)/siNR4A1(s) (**A**) or treated with DIM-C-pPhOH or DIM-C-pPhCO_2_Me (**B**), and ROS was determined using the cell permeable fluorescent probe CM-H2DCFDA as outlined in the Materials and Methods. Rh30 and RD cells were either transfected with siNR4A1 (**C**) or treated with DIM-C-pPhOH or DIM-C-pPhCO_2_Me (**D**), and whole cell lysates were analyzed for TXNDC5, IDH1 and various ER stress genes by Western blot analysis as outlined in the Materials and Methods. (**E**) Rh30 cells were treated with DMSO or 20 μM DIM-C-pPhCO_2_Me, and binding of NR4A1, p300, Sp1 and pol II to the GC-rich regions of the TXNDC5 and IDH1 gene promoters were determined in a ChIP assay as outlined in the 24 hr and (**F**) mRNA levels were determined by real time PCR. Results are expressed as means ± SE (triplicate determinations) and significant (*p <* 0.05) changes in gene expression are indicated.

NR4A1 also regulates expression of *TXNDC5* and *IDH1* to maintain low oxidative and endoplasmic reticulum (ER) stress [16, 18], and transfection of Rh30 and RD cells with siNR4A1 induced ROS as determined using the cell permeable fluorescent indicator CM-H2DCFDA (Figure 5A) and similar results were observed after treatment with the NR4A1 antagonists (Figure 5B). Knockdown of NR4A1 (Figure 5C) or treatment of Rh30 and RD cells with the NR4A1 antagonists (Figure 5D) decreased expression of *TXNDC5* and *IDH1* and this was accompanied by induction of several markers of ER stress including phosphorylated PERK (pPerk), ATF4 and CHOP. Both TXNDC5 and IDH1 have GC-rich promoter sequences at -22 and -112, respectively, in untreated cells, and a ChIP assay showed binding of NR4A1, Sp1 and p300 to the GC-rich regions of the promoter in Rh30 cells (Figure 5E). Treatment of these cells with the NR4A1 antagonist DIM-C-pPhCO_2_Me resulted in decreased interactions of NR4A1, p300 and pol II with the GC-rich TXNDC5 and IDH1 promoters and also some loss of Sp1 from the TXNDC5 promoter, suggesting that like survivin, expression of these genes also involves interaction of the p300/NR4A1 complex with Sp1 at GC-rich elements (Figure 1B). In addition, DIM-C-pPhCO_2_Me also decreased expression of TXNDC5 and IDH1 mRNA levels (Figure 5F). The induction of ROS by inactivation of NR4A1 also has functional significance since DIM-C-pPhOH-induced cleavage of PARP, caspases 3 and 7 (markers of apoptosis), and growth inhibition were significantly reversed after cotreatment with 5 mm glutathione (GSH) (Supplementary Figure S1).

**Figure 6:**
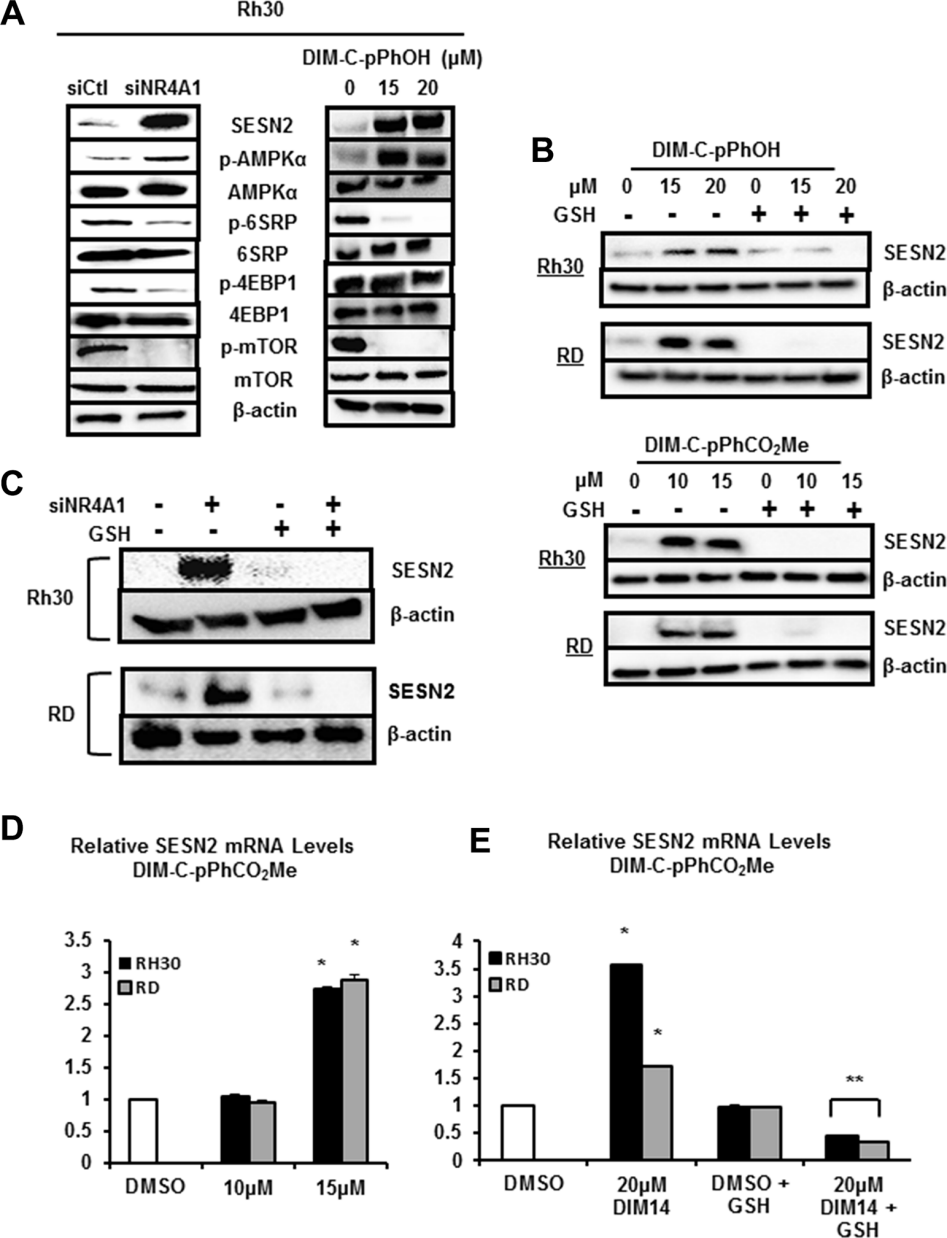
NR4A1 regulates sestrin 2 and mTOR in RMS cells (**A**) Rh30 cells were either transfected with siNR4A1 or treated with DIM-C-pPhOH, and whole cell lysates were analyzed as outlined in the Materials and Methods. Rh30 and RD cells were either treated with DIM-C-pPhOH or DIM-C-pPhCO_2_Me alone or in combination with GSH (**B**) or transfected with siCtl/siNR4A1 alone or in combination with GSH treatment (**C**). Whole cell lysates were analyzed for sestrin 2 (SESN2) by Western blots as outlined in the Materials and Methods. Rh30 and RD cells were treated with DMSO and DIM-C-pPhCO_2_Me alone (**D**) or in combination with GSH (**E**), and expression of sestrin 2 mRNA levels were determined by real time PCR as outlined in the Materials and Methods. Results (D and E) are expressed as means ± SE (3 replicates) and significant (*p <* 0.05) induction (*) or inhibition of induction (**) are indicated.

NR4A1 binds and inactivates p53 (Figure 1B) and knockdown of NR4A1 or treatment with NR4A1 antagonists results in p53-dependent induction of sestrin 2, an upstream regulator of AMPKα in lung and colon cancer cells [12, 16]. Even though Rh30 and RD cells are p53-negative; knockdown of NR4A1 in Rh30 cells or treatment with DIM-C-pPhOH induced sestrin 2 and increased phosphorylation of AMPKα and this resulted in decreased activation of mTOR-dependent phosphorylation of both 4EBP1 and 6SRP which are kinases downstream from mTOR (Figure 6A). Similar results were observed in RD cells (Supplementary Figure S2A) and after treatment with DIM-C-pPhCO_2_Me (Supplementary Figure S2B). Sestrin 2 is also induced in response to ROS [20] and since C-DIM/NR4A1 antagonists induce ROS (Figure 5B), the effects of the antioxidant GSH as an inhibitor of sestrin 2 induction after NR4A1 inactivation was investigated. Sestrin 2 induction in RD and Rh30 cells treated with DIM-C-pPhOH or DIM-C-pPhCO_2_Me was attenuated after cotreatment with GSH (Figure 6B) and similar results were observed after NR4A1 knockdown (Figure 6C). DIM-C-pPhCO_2_Me also induced sestrin 2 gene expression in Rh30 and RD cells (Figure 6D), and the induction response was attenuated in cells cotreated with the antioxidant GSH (Figure 6E). Thus, the NR4A1 antagonists block at least three NR4A1-regulated pro-oncogenic pathways (Figure 1B) in RMS cells indicating that NR4A1 is a potential new drug target for treatment of RMS.

## DISCUSSION

The NR4A orphan nuclear receptors are immediate early genes induced by multiple stressors and there is increasing evidence that these receptors play a critical role in maintaining cellular homeostasis in multiple tissues and organs [8]. There is evidence that NR4A1 is important in metabolism and metabolic disease, cardiovascular and neuronal function, and inflammation in multiple tissues [8]. The function and mechanism of action of NR4A1 in cancer cells is complex; transgenic mice in which both NR4A1 and NR4A3 (Nurr1) have been knocked out rapidly develop an acute myeloid leukemia (AML) type of leukemia and there is evidence that NR4A1 is a tumor suppressor for AML [21, 22]. In contrast, NR4A1 is overexpressed in most solid tumors and is a negative prognostic factor for lung, breast and colon cancer patients and knockdown studies show that NR4A1 plays a role in cancer cell proliferation, survival, migration and invasion [9, 10, 23–27]. Early studies on drug-mediated effects of NR4A1 demonstrated that many apoptosis-inducing drugs that do not directly bind NR4A1 induce nuclear export of this receptor which subsequently binds to mitochondrial bcl-2 to form a pro-apoptotic complex that disrupts mitochondria in cancer cells, resulting in increased cell death (24, 25). However, more recently cytosporone B and some structurally-related compounds have been identified as NR4A1 ligands [14, 15, 17] and studies in this laboratory have also identified C-DIMs as NR4A1 receptor ligands and these compounds act as NR4A1 antagonists that inhibit cancer cell growth and survival by directly targeting nuclear NR4A1 [14–17].

NR4A1 is a nuclear protein expressed in RD and Rh30 cells (Figure 1E), and there is evidence from publically available array data from RMS tumors that NR4A1 mRNA is overexpressed in tumor vs. non-tumor tissue (Figure 1C). The functional role of NR4A1 in RMS was investigated by RNAi showing that this receptor plays a role in RMS cell proliferation and survival (Figures 2 and 3) and these results are comparable to those observed in many other solid tumors [reviewed in 9]. NR4A1 knockdown studies also demonstrated that NR4A1 also plays a role in activating mTOR and maintaining low stress levels through its regulation of TXNDC5 and IDH1 (Figure 5). Both IDH1 and TXNDC5 are regulated by NR4A1 in pancreatic and colon cancer cells, and knockdown of either NR4A1 or TXNDC5 in pancreatic cancer cells results in the induction of ROS [18]. The results illustrated in Figure 5A and 5B show that knockdown of NR4A1 in RMS cells also results in downregulation of TXNDC5 (and IDH1) and induction of ROS and ER stress genes, confirming that NR4A1 regulation of TXNDC5 suppresses ER and oxidative stress. NR4A1 also regulates IDH1 expression which also generates cellular reductants and complements the function of TXNDC5 in terms of stress suppression [20, 28]. In contrast to glioma and other cancer cells which express IDH1 mutations [29] enhancing D-2-hydroxyglutarate production, this mutation has not been detected in RMS [30]. NR4A1-dependent maintenance of low oxidative stress levels also contributes to mTOR signaling since knockdown of NR4A1 results in oxidative stress-dependent induction of sestrin 2 [31] which in turn activates AMPKα and inhibits mTOR. Genomic analysis coupled with high throughput screening of primary RMS cultures identified ROS inducers as a therapeutically relevant approach for treating ERMS [32]. Results of our studies implicate NR4A1 regulation of TXNDC5 and IDH1 for maintaining low oxidative stress in RD and Rh30 cells and suppression of these gene by the C-DIM/NR4A1 antagonists induces ROS which in turn induces ER stress and also sestrin 2-dependent inhibition of mTOR (Figures 5 and 6). Induction of this latter pathway may be an important contributor to the efficacy of C-DIM/NR4A1 antagonists and other ROS-inducing agents since mTOR inhibitors show promise as mechanism-based drugs for RMS chemotherapy [33–35]. Moreover, Supplementary Figure S1 also demonstrates that DIM-C-pPhOH-mediated induction of ROS plays a major role in growth inhibition and induction of apoptosis and this is due to the high sensitivity of RMS cells to ROS-inducing compounds [32].

Nuclear receptors not only activate gene expression through direct binding to their cognate response elements but also indirectly through interactions with DNA-bound transcription factors such as Sp1, and this has been observed for several other nuclear hormone and orphan receptors [36–42]. Similar results have previously been reported for NR4A1 which coactivates expression of growth-promoting and pro-survival genes with GC-rich promoters through interactions of p300/NR4A1-Sp1 bound to GC *cis*-elements [16, 19]. Knockdown of NR4A1 decreases expression of several Sp1-regulated genes (but not Sp1) including *survivin, bcl-2, EGFR, cyclin D1* and c-*Myc* in Rh30 and RD cells as observed in other cancer cell lines and in this study, ChIP assays indicated that not only survivin (Figure 4D) but also TXNDC5 and IDH1 are regulated by interactions of p300/NR4A1 with Sp1 bound to GC-rich promoters (Figure 1B). A previous study reported that Sp1 is overexpressed in RMS tumors and cells and Sp-regulated genes such *platelet-derived growth factor receptor a, hepatocyte growth factor receptor, insulin-like growth factor receptor* and *CXCR4* are important for the oncogenic phenotype of RMS [43]. Current studies using RNA-seq combined with receptor knockdown are focused on the role of NR4A1 in regulating expression of these genes and other pro-oncogenic factors in RMS cells via pathway 3 (Figure 1B).

The important pro-oncogenic functions of NR4A1 in RMS cells indicate that antagonists of this receptor represent a potential novel clinical approach for treating RMS. DIM-C-pPhOH was initially characterized as an inhibitor of NR4A1-dependent transactivation and recent structure-binding studies demonstrate that DIM-C-pPhOH, DIM-C-pPhCO_2_Me and other C-DIMs bind the ligand binding domain of NR4A1 and exhibit NR4A1 antagonist activity in colon cancer cells [16]. In RMS cells, we have also observed parallel effects of NR4A1 knockdown and treatment with DIM-C-pPhOH and DIM-C-pPhCO_2_Me, demonstrating that C-DIM/NR4A1 antagonists represent a new class of mechanism-based drugs for treating RMS. This observation is particularly important for RMS patients since their current treatment regimens rely on cytotoxic drugs which lead to serious health problems later in life [2, 7]

## MATERIALS AND METHODS

### Cell lines, antibodies, chemicals, and other materials

Rh30 and RD human RMS cancer cell lines were obtained from the American Type Culture Collection (Manassas, VA) and were maintained at 37°C in the presence of 5% CO_2_ in RPMI-1640 Medium or Dulbecco's Modified Eagle's Medium, respectively, both supplemented with 10% fetal bovine serum and 5% antibiotic. Dulbecco's Modified Eagle's Medium, and RPMI-1640 were purchased from Sigma-Aldrich (St. Louis, MO), glutathione (GSH) reduced free acid were purchased from Millipore (Temecula, CA), and Lipofectamine 2000 was purchased from Invitrogen (Grand Island, NY). Apoptotic, Necrotic, and Healthy Cells Quantification Kit was purchased from Biotium (Hayward, CA). Cells were subsequently viewed using a filter set for FITC, rhodamine, and DAPI on an Advanced Microscopy EVOS fl, fluorescence microscope. RGB-4103 GelRed nucleic acid stain was used in place of Ethedium Bromide from Phenix Research Products (Candler, NC). The C-DIM compounds were prepared as previously described [16] and a summary of the antibodies are provided in Supplementary Table S1. A summary of oligonucleotide for RNAi and real time PCR and ChIP primers are summarized in Supplementary Table S2.

### Total RNA expression analysis

Patient sample data of total RNA was acquired from NCBI GEO dataset GSE28511 (http://www.ncbi.nlm.nih.gov/geo/query/acc.cgi?acc = GSE28511) and was previously analyzed for quality control, quantile normalized. In addition, multi-probe genes were averaged by the submitter. Expression values were listed into non-tumor and RMS tumor groups in JMP^®^ and a box plot was generated, from which a *t*-test was performed; significance was determined as a *p*-value less than 0.01, shown by an asterisk.

### Cell proliferation and tumor growth assay

Rh30 and RD cells were plated in 12-well plates at 1.0 × 10^5^ and allowed to attached for 24 hr before treatment with DIM-C-pPhOH, DIM-C-pPhCO_2_Me, or transfected with siNR4A1, with DMSO (dimethyl sulfoxide) as empty vehicle or siCtl siRNA (with lipofectamine vehicle) as controls, respectively. Cells were then trypsinized and counted at indicated times using a Coulter Z1 cell counter. Female athymic nude mice (6–8 weeks old) were obtained (Charles River Laboratory, Wilmington, MA) and maintained under specific pathogen-free conditions, and housed at Texas A&M University in accordance with the standards of the Guide for the Care and Use of Laboratory Animals and the Association for Assessment and Accreditation of Laboratory Animal Care (AAALAC). The protocol of the animal study was approved by the Institutional Animal Care and Use Committee, Texas A&M University. Rh30 cells (4 × 10^6^ cells) grown in RPMI media containing 10% FBS were detached, resuspended in 100 μl of phosphate-buffered saline with matrigel (BD Bioscience, Bedford, MA) (75:25), and implanted subcutaneously in the mice. When tumors reached about 40–50 mm^3^ size, the animals were randomized into control and treatment groups (6 animals per group) and mice were treated with placebo or DIM-C-pPhCO_2_Me (40 mg/kg/d) in corn oil by oral gavage every second day for 20 days. Tumor volumes and weights, and body weight were determined; the tumor size was measured using Vernier calipers, and the tumor volume was estimated by the formula: tumor volume (mm^3^) = (L × W^2^) × ½, where L is the length and W is the width of the tumor.

### Annexin V staining

Rh30 and RD cells were seeded in 2-well Lab-Tek chambered B#1.0 Borosilicate coverglass slides from Thermo Scientific and were allowed to attach for 24 hr before treatment with C-DIMs or DMSO for 48 hr and with siNR4A1 (100 μM) or siCtl for 72 hr, and Annexin V staining was determined as described [16].

### Immunofluorescence

Rh30 and RD cells were plated in seeded at 1.0 × 10^5^ in 2-well Lab-Tek chambered B#1.0 Borosilicate coverglass slides from Thermo Scientific and were allowed to attach for 24 hr in DMEM/Ham F-12 containing 5.0% charcoal-stripped fetal bovine serum and treated with C-DIM compounds for 24 hr. Cells were then treated with fluorescent NR4A1 antibody [Nurr77 (D63C5) XP^®^] and the manufacturer's protocol (Cell Signaling Technologies, Danvers, MA) was used to observe immunofluorescence. Hoechst staining from the apoptotic and necrotic cells assay (Biotium, Hayward, CA) was used to visualize nuclear DAPI staining, while NR4A1 localization was determined by green fluorescence. Images were taken using an EVOS fluorescence microscopy from Advance Microscopy; NR4A1 and DAPI images were subsequently merged.

### Western blot

Rh30 and RD cells were seeded in 6-well plates at 1.0 × 10^5^ and allowed to attached for 24 hr before treatment with DIM-C-pPhOH, DIM-C-pPhCO_2_Me, or transfected with siNR4A1, with DMSO as empty vehicle or siCtl siRNA (with lipofectamine vehicle) as controls, respectively. Cells were treated with C-DIMs or DMSO for 48 hr or transfected with siNR4A1 (100 μM) or siCtl for 72 hr, and Western blots of whole cell lysates were determined as described [16].

### Transactivation, real-time PCR, and chromatin immunopricipitation (ChIP) assays

Real time PCR and ChIP assays using RMS cell lines transfected with oligonucleotides or treated with C-DIMs were carried out essentially as described [12, 16, 18, 19], and the oligonucleotides and primers used are summarized in Supplementary Table S2. Transactivation studies were carried out in RD cells transfected with two NR4A1-responsive constructs, NuRE_×3_-luc and NBRE_3_-luc, that bind NR4A1 as a homodimer or monomer, respectively, or transfected with a GAL4-NR4A1 (chimera) and a GAL4-responsive construct (UAS_×5_-luc) essentially as described [44]. Real-time PCR and chromatin immunoprecipitation assays were carried out essentially as described [19].

### Generation and measurement of ROS

Cellular ROS levels were measured utilizing a cell permeable probe, CM-H2DCFDA (5-(and-6)-chloromethyl-2′7′-dichlorodihydrofluorescein diacetate acetyl ester) from Invitrogen (Grand Island, NY). CM-H2DCFDA diffuses into the cell, where its acetate groups are cleaved by intracellular esterases and upon oxidation, yields a fluorescent adduct that is measured by flow cytometry using Accuri's C6 Flow Cytometer (Ann Arbor, MI). Cells were plated in a 6-well culture plate and allowed to attach for 24 hr and treated for the indicated time with DIM-C-pPhOH, DIM-C-pPhCO_2_Me, or siNR4A1. Subsequently, cells were trypsinized, neutralized, then loaded with 10 μM of probe for 20 min incubation, and were washed with serum free media for ROS quantification.

### Statistics

Results for each treatment group were replicated (at least 3X) and expressed a means ± SE. Statistical comparisons of the treated groups vs. a control for each treatment were determined using Student's *t*-test.

## SUPPLEMENTARY MATERIALS FIGURES AND TABLES


