# Supplementary material for: Nuclear receptor 4A1 (NR4A1) as a drug target for treating rhabdomyosarcoma (RMS)
Source: Oncotarget. 2016 Apr 29;7(21):31257–69. doi: 10.18632/oncotarget.9112 (PMC5058754; doi:10.18632/oncotarget.9112)
Supplement: Supplementary file 1 [file oncotarget-07-31257-s001.pdf]

Nuclear receptor 4A1 (NR4A1) as a drug target for treating rhabdomyosarcoma (RMS)

Supplementary Materials

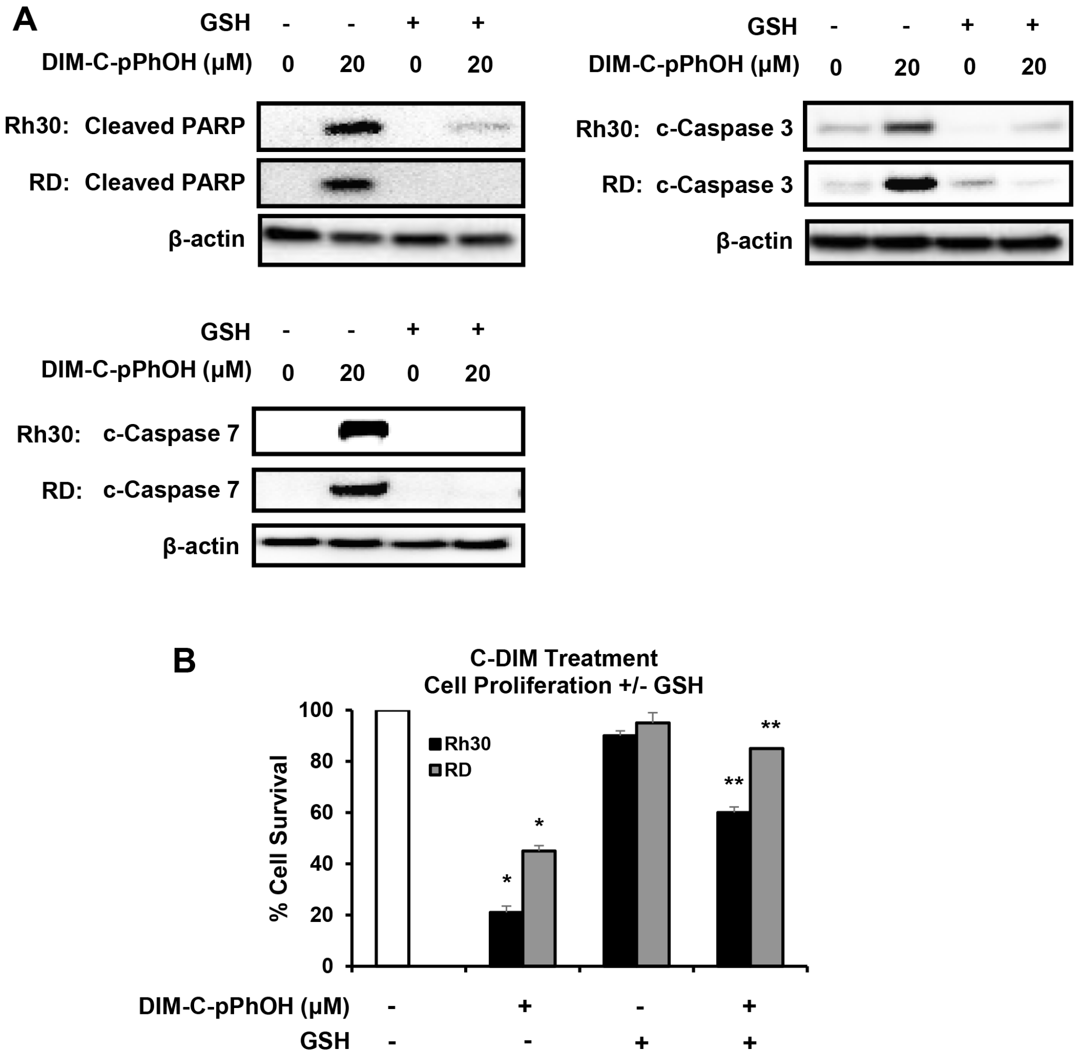

Supplementary Figure S1: (**A**) Apoptosis. Cells were treated with DIM-C-pPhOH, glutathione (GSH) alone and in combination for 24 hr, and whole cell lysates were analyzed by western blots. (**B**) Growth Inhibition. Cells were treated as described in (A) and cell growth was determined. [Significant ($p < 0.05$) inhibition (*) and reversal (**).

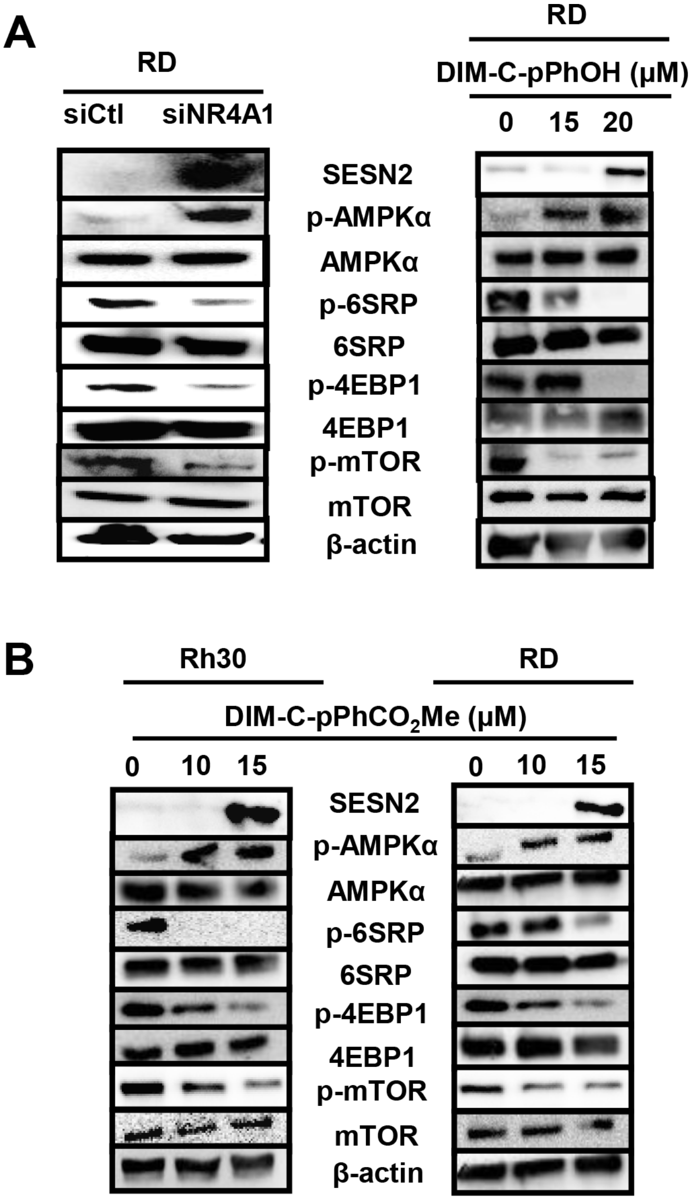

Supplementary Figure S2: RD cells were transfected with siCtl or siNR4A1 or treated with DIM-C-pPhOH (A) and both RMS cell lines were treated with DIM-C-pPhCO2Me (B) and whole cell lysates were analyzed by Western blots as outlined in the Materials and Methods.

Supplementary Table S1: Antibodies

Antibodies	Vendors
Phospho PERK	Biolegend (San Diego, CA).
Sp1 antibody	Millipore (Temecula, CA)
SESN2	Santa Cruz (Santa Cruz, CA)
Bcl-2	Santa Cruz (Santa Cruz, CA)
CHOP	Santa Cruz (Santa Cruz, CA)
ATF	Santa Cruz (Santa Cruz, CA)
IDH1	Santa Cruz (Santa Cruz, CA)
P300	Santa Cruz (Santa Cruz, CA)
EGFR	Santa Cruz (Santa Cruz, CA)
Normal IgG	Santa Cruz (Santa Cruz, CA)
Cyclin D1	Abcam (Cambridge, MA)
TXNDC5	GeneTex (Irvine, CA)
RNA Polymerase II	GeneTex (Irvine, CA)
TXNDC5	GeneTex (Irvine, CA)
NR4A1	Cell Signaling Technologies (Danvers, MA)
c-PARP	Cell Signaling Technologies (Danvers, MA)
Survivin	Cell Signaling Technologies (Danvers, MA)
cMyc	Cell Signaling Technologies (Danvers, MA)
p/AMPKα	Cell Signaling Technologies (Danvers, MA)
p/S6RP	Cell Signaling Technologies (Danvers, MA)
p/4EBP1	Cell Signaling Technologies (Danvers, MA)
p/mTOR	Cell Signaling Technologies (Danvers, MA)
β-actin	Sigma Aldrich (St. Louis, MO)

Supplementary Table S2: Primers and oligonucleotides

Primers

Survivin promoter	5′-TCC AGG ACT CAA GTG ATG CTC-3′ (sense)
	5′-TCA AAT CTG GCG GTT AAT-3′ (antisense)
TXNDC5 promoter	5′-CTC GCT CCA GCC CTT CCC TG-3′ (sense)
	5′-AGC AGC AGC AGC AGC GCA GTC A-3′ (antisense)
IDH1 promoter	5′-TTA CAT GGT TGA TGC GGC TT-3′ (sense)
	5′-GCC TAA TCT CGG CCA AAA GA-3′ (antisense)
Sestrin	5′-GGC ACT TCC GCC ACT CA-3′ (sense)
	5′-TCA GGT CAT GTA GCG GGT G-3′ (antisense)
Cyclin D1	5′-ACA AAC AGA TCA TCC GCA AAC AC-3′ (sense)
	5′-TGT TGG GGC TCC TCA GGT TC-3′ (antisense)
Survivin	5′-GCC CAG TGT TTC TTC TGC TT-3′ (sense)
	5′-CCG GAC GAA TGC TTT TTA TG-3′ (antisense)
EGFR	5′-TGC GTC TCT TGC CGG AAT-3′ (sense)
	5′-GGC TCA CCC TCC AGA AGG TT-3′ (antisense)
TXNDC5	5′-GGG TCA AGA TCG CCG AAG TA-3′ (sense)
	5′-GCC TCC ACT GTG CTC ACT GA-3′ (antisense)
IDH1	5′-AAG GAT GCT GCA GAA GCT ATA A-3′ (sense)
	5′-CCA TAA GCA TGA CGA CCT ATG A-3′ (antisense)

Oligonucleotides

siCtl	CGU ACG CGG AAU ACU UCG A
siNR4A1(1)	SASI_Hs02_00333289
siNR4A1(2)	SASI_Hs01_00182072

*All primers and oligonucleotides were purchased from Sigma Aldrich (St. Louis, MO).
